# A qPCR Assay to Detect and Quantify Shiga Toxin-Producing *E. coli* (STEC) in Cattle and on Farms: A Potential Predictive Tool for STEC Culture-Positive Farms

**DOI:** 10.3390/toxins6041201

**Published:** 2014-03-27

**Authors:** Karen Verstraete, Els Van Coillie, Hadewig Werbrouck, Stephanie Van Weyenberg, Lieve Herman, Jurgen Del-Favero, Peter De Rijk, Lieven De Zutter, Maria-Adelheid Joris, Marc Heyndrickx, Koen De Reu

**Affiliations:** 1 Technology and Food Science Unit, Institute for Agricultural and Fisheries Research (ILVO), Brusselsesteenweg 370, Melle 9090, Belgium; E-Mails: karen.verstraete@ilvo.vlaanderen.be (K.V.); els.vancoillie@ilvo.vlaanderen.be (E.V.C.); hadewig.werbrouck@ilvo.vlaanderen.be (H.W.); stephanie.vanweyenberg@ilvo.vlaanderen.be (S.V.W.); lieve.herman@ilvo.vlaanderen.be (L.H.); marc.heyndrickx@ilvo.vlaanderen.be (M.H.); 2 Applied Molecular Genomics Group, Department of Molecular Genetics, Flemish Institute for Biotechnology (VIB), Universiteitsplein 1, Antwerpen 2610, Belgium; E-Mails: jurgen.delfavero@molgen.vib-ua.be (J.D.-F.); Peter.DeRijk@molgen.vib-ua.be (P.D.R.); 3 Department of Veterinary Public Health and Food Safety, Ghent University, Salisburylaan 133, Merelbeke 9820, Belgium; E-Mails: lieven.dezutter@ugent.be (L.D.Z.); adelheid.joris@gmail.com (M.-A.J.); 4 Department of Pathology, Bacteriology and Poultry Diseases, Ghent University, Salisburylaan 133, Merelbeke 9820, Belgium

**Keywords:** Shiga toxin, *E. coli*, real-time PCR, cattle, quantification, intimin, screening, farm, feces, isolation

## Abstract

Shiga toxin-producing *E. coli* (STEC), of various serogroups harboring the intimin gene, form a serious threat to human health. They are asymptomatically carried by cattle. In this study, a quantitative real-time PCR (qPCR) method was developed as a molecular method to detect and quantify Shiga toxin genes *stx1* and *stx2* and the intimin gene *eae*. Subsequently, 59 fecal samples from six farms were tested using qPCR and a culture method as a reference. Three farms had contaminated animals as demonstrated by the culture method. Culture-positive farms showed moderate significantly higher *stx* prevalences than culture-negative farms (*p* = 0.05). This is the first study which showed preliminary results that qPCR can predict STEC farm contamination, with a specificity of 77% and a sensitivity of 83%, as compared with the culture method. Furthermore, the presence or quantity of *stx* genes in feces was not correlated to the isolation of STEC from the individual animal. Quantitative data thus did not add value to the results. Finally, the detection of both *stx* and *eae* genes within the same fecal sample or farm using qPCR was not correlated with the isolation of an *eae*-harboring STEC strain from the respective sample or farm using the culture method.

## 1. Introduction

Shiga toxin-producing *Escherichia coli* (STEC) are important food-borne pathogens which can cause severe disease, including hemorrhagic colitis (HC) and a life-threatening complication known as hemolytic uremic syndrome (HUS) [1,2]. The prominent virulence factor of STEC is the phage-encoded Shiga toxin 1 or 2 (*stx1* or *stx2* genes), which is responsible for kidney failure in humans. The adhesin intimin (*eae* gene) is often present in human pathogenic strains, where it mediates both intimate attachment to the intestinal epithelial cells, as well as lesions (both attaching and effacing) in the intestinal mucosa. *eae* is also carried by EPEC (Enteropathogenic *E. coli*), a pathotype of *E. coli* that can cause diarrhea in humans but for which no zoonotic transmission route exists [3]. For each of these genes, different subtypes were described, showing variation in DNA and amino acid sequence [4]. For intimin, the allelic differentiation mediates host tissue tropisms, whereas for the Shiga toxins it also involves different biological activity with a correlation to the clinical manifestations [5,6]. Additionally, virulence factors are transferrable between microorganisms, especially those encoded on mobile elements like plasmids and bacteriophages [7]. Human pathogenic STEC strains mainly belong to the serotypes O157:H7, O26:H11, O103:H2, O111:H8, and O145:H28 [8], which generally possess the *eae* gene. Of these, serotype O157:H7 has been the one studied most extensively.

Domestic ruminants, mainly cattle, have been implicated as the principal reservoir of the STEC pathogens [9]. They can carry both STEC O157 and non-O157 serogroups. Cattle play an important role in the epidemiology of human infections, because food contaminated with cattle feces is the most prominent contamination source [10]. On-farm control of the pathogen first requires a thorough understanding of on-farm epidemiology. Both prevalence data and quantitative data are important for epidemiology [11]. Either culture or molecular methods can be used. Culture methods have the disadvantage of generally targeting only a subset of serogroups [12]. Furthermore, they are labor-intensive and time-consuming. A culture-dependent method was developed to simultaneously isolate this subset of five serogroups by using selective agars [13]. Quantification of STEC strains using a culture method is possible using either the most probable number (MPN) technique or direct plating [14]. The disadvantages of culture methods led us to develop and evaluate a quantitative real-time PCR (qPCR) method. In the literature, qPCR methods have been described for the detection [15] and quantification [16,17,18] of STEC genetic markers in cattle feces. Ibekwe *et al.* [17] explored the potential of qPCR to quantify STEC O157 in naturally contaminated cattle feces; they promote a culture-free approach.

The aim of this study was to investigate if a molecular method can predict STEC contamination of cattle or cattle farms as compared to a culture method. A molecular method is DNA-based and can only detect the genetic material of STEC. In order to determine the actual presence of STEC or *eae*-harboring STEC, a culture method is always needed. Therefore, the qPCR method was evaluated with a culture method as a reference. Second, we evaluated if quantitative data obtained by qPCR would give additional information about the degree of contamination of the individual animal or farm. Third, we evaluated if *stx* and *eae* were detected in the same sample by qPCR, then could *eae-*harboring STEC be isolated from the respective sample by means of the culture method? For all of these purposes, a quantitative real-time PCR method was developed which targeted most clinical variants of *stx1*, *stx2*, and *eae*. Primers and probes needed to be optimal, as all variants of *stx* and *eae* needed to be detected with the same sensitivity.

## 2. Results

### 2.1. Method Development and Testing

The utility of the selected primers was evaluated in qPCR assays using SYBR Green I and DNA standards of strains MB3936 and MB4378. For *stx1* quantification, C_q_ values for 10^5^ gene copies were lower for the newly designed primers (stx1-F/-R; C_q_ 23.0) than for the primers adapted from literature (598-F/1015’-R; C_q_ 30.0 and 598-F/1015”-R; C_q_ 28.3). Also, results of the slopes of the standard curves showed that the newly designed primer pairs were preferable (slope close to −3.34). Likewise for *stx2*, the newly designed primers gave lower C_q_ values for 10^5^ copies (stx2-F/-R; C_q_ 28.0) than the primers adapted from literature (C_q_ 30.9) and slopes were close to −3.34. However, to make the C_q_ value of the newly designed primer set for *stx2* comparable to that used for *stx1*, the inosine to compensate for one of the two polymorphisms in the sequence of the reverse primer was replaced by the respective specific base, leaving only one per primer (stx2-R_a_ and stx2-R_b_). As a consequence, C_q_ values for 10^5^ copies dropped drastically (stx2-F/-R_a_; C_q_ 22.3 and stx2-F/-R_b_; C_q_ 22.7) compared to the original primer set. This resulted in quantification of *stx2* using two primer sets, a and b. For *eae*, both newly designed primer pairs gave similar low C_q_ values for 10^5^ copies (eae-F/-R; C_q_ 23.5 and eae-F/-R_2_; C_q_ 23.1) and slopes were close to −3.34.

qPCR assays were performed using the final selection of primers (Table 1) with corresponding probes together with the appropriate DNA standards of strain MB3936 or MB4378. Properties of the standard curves are listed in Table 2.

For *stx2* using primer-probe set a, positive no-template controls were consistently found after 36 cycles. Consequently, a cut-off value was set at C_q_ 36. Therefore, the lowest number of gene copies detected with this primer-probe set was between 10 and 100 copies per reaction tube.

Inhibition was evaluated on eight cattle fecal samples that were previously demonstrated to be negative using qPCR and culture method. Cq values did not increase as compared to the standard curve, pointing to an absence of inhibition (data not shown).

**Table 1:** Primers and probes designed in this study for qPCR quantification of STEC. Corresponding nucleotide positions in the sequences of indicated EMBL/Genbank accession numbers are given.

Gene	Primer or probe ^∂^	Sequence (5’- 3’) ^†^	Position (5’- 3’)	Accession number
*stx1*	stx1-F	GAC GCA GTC TGT IGC AAG AG	516-535	Z36899
	stx1-R	cga aaa cgi aaa gct tca gct g	581-560	Z36899
	stx1-P ^Ф^	ATG TTA CGG TTT GTT ACT GTG	538-558	Z36899
*stx2*	stx2-F	TCA GGC AIA TAC AGA GAG AAT TTC G	578-602	AY443044
	stx2-R_a_	ccg gig tca tcg tat aca cag	646-626	AY443044
	stx2-R_b_	ccg gig tca tcg tat aaa cag	646-626	AY443044
	stx2-P ^Ф^	CAC TGT CTG AAA CTG CT	608-624	AY443044
*eae*	eae-F	GGA AGC CAA AGC GCA CAA	1507-1524	AF025311
	eae-R	ggc icg agc igt cac ttt ata a	1593-1572	AF025311
	eae-P ^§^	TAC CAG GCT ATT TTG CCI GCT TAT GTG C	1528-1555	AF025311

Notes: ^∂^ Forward primers with suffix -F; Reverse primers with suffix -R; Probes with suffix -P. ^Ф^ Probe tagged with minor groove-binding non-fluorescent quencher (MGBNFQ) and 6-carboxyfluorescin (FAM) fluorescent label (Applied Biosystems). ^§^ Probe tagged with black hole quencher (BHQ-1) and a FAM fluorescent label (Eurogentec).

**Table 2:** Properties of the standard curves of the qPCR assays for *stx1*, *stx2*, and *eae* detection and quantification.

Target gene	LOQ ^∂^ (copies/reaction)	Cq ^Ф^ for 10^5^ copies/reaction	Efficiency	R^2^ (Regression coefficient)
*stx1*	1 to 10	24.3	90%	0.9997
*stx2* (using primer set a)	10 to 100	22.8	94%	0.9983
*stx2*(using primer set b)	1 to 10	23.9	87%	0.9997
*eae*	1 to 10	23.9	98%	0.9992

Notes: ^∂^ Limit of quantification; ^Ф^ Threshold cycle for qPCR gene detection and quantification.

**Figure 1:**
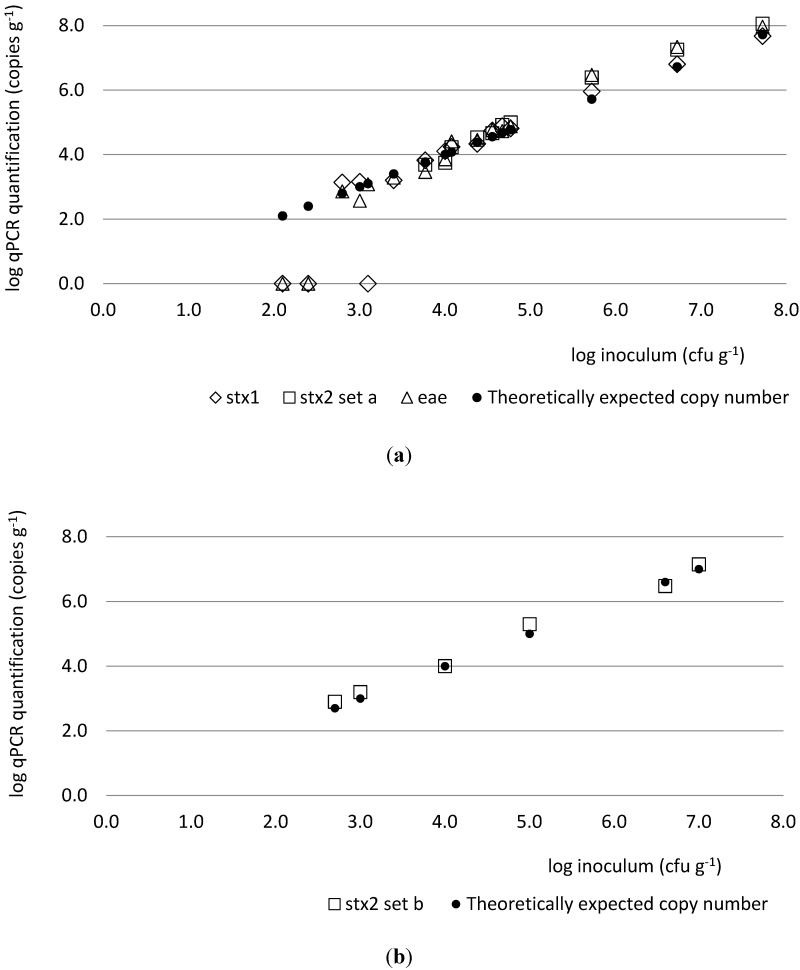
Quantification of *stx1*, *stx2* and *eae* genes by qPCR in cattle fecal samples artificially inoculated with STEC cells. Artificial inoculation was performed using various dilutions of strain MB3936 in 16 contamination levels (**a**) and of strain MB4378 in five contamination levels (**b**). Primer-probe set a was used to quantify *stx2* gene of strain MB3936 and primer-probe set b to quantify *stx2e* gene of strain MB4378.

The limit of quantification (LOQ) was <2.7 log copies g^−1^ feces for *stx1*, *stx2* (primer-probe set b) and *eae*, and <3.7 log copies g^−1^ feces for *stx2* (primer-probe set a) (Figure 1a). The quantification of genes did not differ more than 1 log from the theoretically calculated number based on the inoculums of strain MB3936 or MB4378 for concentrations ≥2.7 log CFU g^−1^ for *stx1*, *stx2* (primer-probe set b) and *eae*, and for concentrations ≥3.7 log CFU g^−1^ for *stx2* (primer-probe set a) (Figure 1b). The reference sample (blank) was found negative for *stx1*, *stx2* and *eae*.

Results on inclusivity and exclusivity are listed in Table 3. *E. coli* strains carrying different variants of the genes *stx1*, *stx2* and *eae* were detected using the qPCR assays, except for one strain carrying *stx2f,* which was not targeted by the qPCR assays. No amplification was noticed for any of the non-*E. coli* strains, the non-pathogenic *E. coli* type strain, or the ETEC strain, which did not carry *stx* and/or *eae* genes. For *Shigella dysenteriae, Citrobacter rodentium*, and *Escherichia albertii,* qPCR detected *stx1* or *eae* genes.

**Table 3:** Bacterial strains used to test inclusivity (EPEC and STEC strains) and exclusivity (non-pathogenic *E. coli* or non-*E. coli* strains) of the qPCR assays for *stx1*, *stx2* and *eae* and the respective results.

Species orbacterial subgroup	Strain	Serotype	Virulence genes			qPCR detection		
			*stx1*	*stx2*	*eae*	*stx1*	*stx2*	*eae*
EPEC ^∂^	MB3885 ^†^	O157	-	-	*eae γ1*	-	-	+
	MB3886	O157	-	-	*eae γ1*	-	-	+
STEC ^Ф^	MB3892	O91	*stx1ab*	*stx2b*	-	+	+	-
	MB3900	O175	-	*stx2*	-	-	+	-
	MB3957	O146	*stx1ab, stx1c*	*stx2b*	-	+	+	-
	MB3963	O128ab	*stx1ab, stx1c*	*stx2b*	-	+	+	-
	MB3986	O181	*stx1ab*	-	-	+	-	-
	MB4213	no info	-	*Stx2d, stx2e, stx2g*	-	-	+	-
	MB4376 (EH250)	O118	-	*stx2b*	-	-	+	-
	MB4377	no info	*stx1d*	-	-	+	-	-
	MB4378	O138	-	*stx2e*	-	-	+	-
	MB4380	no info	*stx1c*	*stx2b*	-	+	+	-
	MB3893	O145	*stx1ab*	-	*eae γ1*	+	-	+
	MB3920	O157	-	*stx2*	*eae γ1*	-	+	+
	MB3936	O26	*stx1ab*	*stx2*	*eae β1*	+	+	+
	MB3938	O145	-	*stx2d*	*eae γ1*	-	+	+
	MB4033	O111	*stx1ab*	*stx2*	*eae γ2*	+	+	+
	MB4074	O26	*stx1ab*	-	*eae β1*	+	-	+
	MB4108	O111	*stx1ab*	*stx2*	*eae γ2*	+	+	+
	MB4117	O103	*stx1ab*	-	*eae ε*	+	-	+
	MB4141	O103	*stx1ab*	*stx2d*	*eae ε*	+	+	+
	MB4208	O157	*stx1ab*	*stx2c*	*eae γ1*	+	+	+
	MB4379	Orough	-	*stx2f*	*eae*	-	-	+
ETEC ^§^	MB1520	-	-	*-*	-	-	-	*-*
*Escherichia coli*	MB544 (LMG2092^T^)	-	-	*-*	-	-	-	*-*
*Shigella dysenteriae*	MB 4436 (CIP 57.28)	-	*stx1ab*	*-*	-	+	-	-
*Citrobacter rodentium*	MB4471 (ATCC 51116)	-	-	*-*	*eae*	-	-	+
*Escherichia albertii*	MB4434 (LMG 20972)	-	-	*-*	*eae*	-	-	+
*Enterobacter aerogenes*	MB260	-	-	*-*	*-*	-	-	*-*
*Citrobacter diversus*	MB423	-	-	*-*	*-*	-	-	-
*Hafnia alvei*	MB291	-	-	*-*	-	-	-	*-*
*Klebsiella pneumoniae*	MB263	-	-	*-*	-	-	-	*-*
*Salmonella* Dublin	MB1145	-	-	*-*	-	-	-	*-*
*Salmonella* Typhimurium	MB1135	-	-	*-*	-	-	-	*-*
*Serratia proteamaculans*	MB262	-	-	*-*	-	-	-	*-*
*Shigella boydii*	MB4435	-	-	*-*	-	-	-	-
*Yersinia enterocolitica*	MB868	-	-	*-*	-	-	-	*-*
*Campylobacter jejuni*	MB1263	-	-	*-*	-	-	-	*-*
*Pseudomonas aeruginosa*	MB289	-	-	*-*	-	-	-	*-*
*Bacillus subtilis*	MB3611	-	-	*-*	-	-	-	*-*
*Clostridium perfringens*	MB128	-	-	*-*	-	-	-	*-*
*Enterococcus faecalis*	MB30	-	-	*-*	-	-	-	*-*
*Listeria monocytogenes*	MB38	-	-	*-*	-	-	-	*-*
*Staphylococcus aureus*	MB4038	-	-	*-*	-	-	-	*-*
*Streptococcus thermophilus*	MB1654	-	-	*-*	-	-	-	*-*

Notes: ^∂^ EPEC, Enteropathogenic *Escherichia coli*; ^Ф^ STEC, Shiga toxin-producing *Escherichia coli*; ^§^ ETEC, Enterotoxigenic *Escherichia coli*; ^†^ Strains with the MB collection number belong to the collection of ILVO’s Technology and Food Science Unit (ILVO-T&V), Laboratory of Molecular Bacteriology. EPEC and STEC strains were kindly donated by the Belgian national VTEC reference laboratory (by D. Piérard).

### 2.2. Study of Molecular Method on Native Cattle Fecal Samples

**Figure 2:**
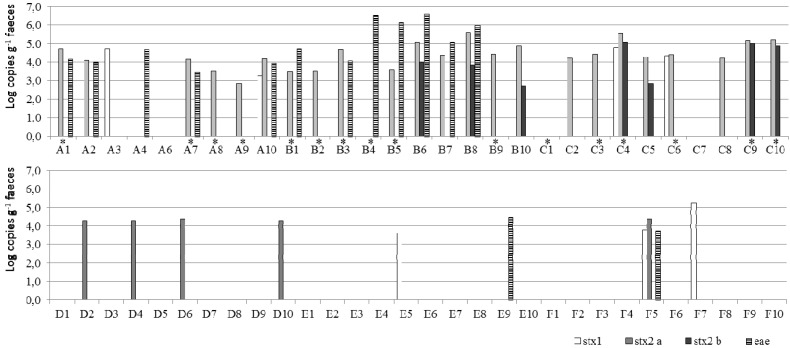
Quantification of *stx1*, *stx2* and *eae* genes by qPCR in individual cattle fecal samples (*n* = 59). (**a**) Samples originated from three culture-positive farms (A, B, C) and (**b**) three culture-negative farms (D, E, F). * Cattle fecal samples that were found to be culture-positive. Primer-probe sets a and b were used to quantify all possible *stx2* variants.

Using the culture method, STEC strains were isolated from 10 animals originating from three farms (farm A, B, and C; Table 4). These were named the culture-positive farms. On farms D, E, and F, no STEC strains could be isolated from any of the animals tested. Using the molecular qPCR method, *stx* (*stx1* and/or *stx2*) was detected in 78%, 90%, and 80% of the animals tested on farms A, B, and C, respectively (Figure 2). On farms D, E, and F, *stx* was detected in 40%, 10%, and 20% of the animals tested, respectively. Statistical analysis showed that the prevalence of *stx* was moderate significantly higher on the culture-positive farms (A, B, C) than on the culture-negative farms (D, E, F) (*p* = 0.05).

**Table 4:** PCR characterization of STEC isolates recovered from cattle fecal samples carrying STEC virulence genes.

Sample	Farm	Serogroup ^∂^	Virulence gene isolate			Enumeration of virulence genes in fecal sample (log copies g^−1^)			
						*stx1*	*stx2* (using primer set a)	*stx2b* (using primer set b)	*eae*
A1	A	O157	*stx1*	*stx2*	*eae*	0.0	4.7	0.0	4.2
A7	A	O157	*stx1*	*stx2*	*eae*	0.0	4.2	0.0	3.5
A8	A	O157	*stx1*	*stx2*	*eae*	0.0	3.5	0.0	0.0
A9	A	O157	*stx1*	*stx2*	*eae*	0.0	2.8	0.0	0.0
B9	B	*-*	*-*	*stx2*	*-*	0.0	4.4	0.0	0.0
C1	C	*-*	*stx1*	*stx2*	*-*	0.0	0.0	0.0.	0.0
C3	C	*-*	*-*	*stx2*	*-*	0.0	4.4	0.0	0.0
C4	C	O26	*stx1*	*-*	*eae*	4.8	5.6	5.1	0.0
C6	C	O26	*stx1*	*-*	*eae*	4.3	4.4	0.0	0.0
C9	C	O26	*stx1*	*-*	*eae*	0.0	5.2	5.0	0.0

Note: ^∂^ Serogroups targeted by PCR include O26, O91, O103, O111, O121, O145, and O157.

Cross tabulation of the qPCR detection of *stx* in the individual animal *vs.* the culture-positive or culture-negative status of the farm showed that the qPCR technique has the potential of being a good predictive screening test for STEC culture-positive farms (Table 5), with a McNemar’s coefficient of 0.774, and a Kappa value of 0.60. The specificity was 77% and the sensitivity 83% at farm level in comparison to the culture method. A specificity of 77% indicates the likelihood of finding an *stx*-negative animal as determined by qPCR on a STEC culture-negative farm. A sensitivity of 83% indicates the likelihood of finding an *stx*-positive animal on a STEC culture-positive farm.

**Table 5:** Cross tabulation of qPCR *stx* detection in cattle fecal samples *versus* the culture-positive or culture-negative status of its respective farm.

		Result culture method:STEC culture-positive farm		Total
		Negative	Positive	
Result qPCR method: *stx* detection in the individual fecal sample	Negative	23	5	28
	Positive	7	24	31
	Total	30	29	59

At the level of the individual animal, the presence of stx was not correlated to the isolation of STEC from the respective fecal sample. This was shown by a McNemar’s coefficient of 0.000 and a Kappa value of 0.009. STEC was isolated from 10 animals. These animals originated from three farms. Three farms were thus culture-positive and three were culture-negative. In total, 31 animals carried stx genes in their feces as determined with qPCR, of which 24 animals were located on culture-positive farms. However, STEC strains could be isolated using the culture method from only nine stx-positive animals as determined by qPCR; no STEC strains could be isolated from the other 15 stx-positive animals, and furthermore, no STEC strains could be isolated from the seven stx-positive samples at the culture-negative farms. Moreover, a STEC strain could be isolated from one stx-negative animal as determined with qPCR. All these findings demonstrate that there was no correlation between the presence of stx as determined with qPCR and the isolation of STEC from the respective sample.

In addition, the virulence genes carried by the isolate (Table 4) did not correspond to the genes detected in the sample as measured by qPCR (Figure 2). For example, the STEC O157 strains isolated from farm A all contained *stx1*, but when using qPCR, the *stx1* gene could not be detected in the fecal sample. The same was observed for the *eae* gene for two out of the four samples. Conversely, on farm C, the *stx2* gene was detected in the sample using qPCR, but the STEC strains that were isolated did not carry this gene.

Because *stx* detection was not related to the isolation of STEC in the individual animal, quantitative data were also not informative about the contamination level of the animal. Quantitative data of *stx* (*stx1* and *stx2*) were also not related to the contamination status of the farm, as the level of *stx* in qPCR-positive samples was in the same range (between ≤ 5 × 10^2^ and 4 × 10^5^ copies g^−1^) for culture-positive and culture-negative farms (Figure 2).

No correlation was found between a combined presence of *stx* and *eae* genes in the animal feces as determined by qPCR and the isolation of *eae*^−^ harboring STEC strains from the respective individual animal or farm by the culture method (Figure 2). In detail, *eae* and *stx* genes were detected within the same cattle fecal samples that did not harbor *eae*^+^ STEC strains and *vice versa*. On the farm level, *eae*^−^ harboring STEC strains were isolated from farms A and C (Table 4). On these farms, prevalences of *stx* and eae were 78 and 44%, respectively, for farm A and 80, and 0%, respectively for farm C (Figure 2). On farm B, no *eae*^−^ harboring STEC strains were isolated, but the prevalence of *stx* and *eae* were 90% and 70%, respectively.

Within a sample, quantification levels of *stx1*, *stx2* and *eae* were frequently unequal (Figure 2).

## 3. Discussion

This report describes the development of a qPCR method to quantify STEC virulence genes *stx1*, *stx2* and *eae* in cattle feces. To the best of our knowledge, this is the first study in which the level of these genes in correlation to the isolation of STEC strains on the respective farm has been evaluated. In literature, a correlation has been demonstrated between high-level shedding of *E. coli* O157 and high levels of *stx* genes in that animal’s feces [16,17]. However, in these studies, only a small number of native samples were tested, with the focus on serogroup O157 only; no attention was given to culture-negative samples nor to the contamination status of the respective farm. In this study, the value of qPCR was studied using both quantitative and qualitative data. We used a serogroup-independent approach in which both the *stx* and *eae* genes were included as genetic markers of virulence.

In terms of method development, all variants of the genes which have been implicated in human disease, except *stx2f*, were included for primer design to make this study relevant to risk assessment of cattle farms for public health. Subtype *stx2f* was genetically too divergent to include in the assay, and it has only rarely been associated with a clinical case [19]. For subtypes *stx2a* and *stx2c*, reports indicate that they have been found most often in HUS cases, with *stx2d* and *stx2e* found less frequently [6], and *stx2g* has never been found, despite its cytotoxicity for Vero cells [20]. They all form a potential risk for human health and were thus all targeted in the current assay. 

We aimed for 100% matching of primers to the annealing sites of the target gene. This ensured optimal efficiency of the PCR and excluded quantitative underestimation of the target [21]. During method development, we observed that a single base mismatch resulted in a log 3 reduction in the gene copy number. This is detrimental for reliable quantification. We demonstrated above that most of the primers and probes described in the literature for STEC detection or quantification [17,22,23,24,25,26,27,28,29,30,31] contained several mismatches when aligned to gene sequences of the different variants of *stx1*, *stx2* and *eae*. This implies that amplification is not optimal for some gene variants only. For primer-probe sets that contained few mismatches (≤2 in a primer, ≤1 in a probe) [32,33], inosine bases were built in and the qPCR efficiency was evaluated. Preliminary results demonstrated that primers designed in this study were the most efficient compared to those already published, despite needing two primer sets for *stx2* quantification. Corresponding probes for *stx1* and *stx2* contained a minor groove-binding tag which heightens the melting temperature (Tm) of the probe by attaching to the minor groove of the target DNA. This enables the probe to be made shorter. This is useful in cases where conserved regions are limited in length. Notwithstanding the drawback of positive no-template controls for one primer pair (stx2-F/-R_a_) and the subsequent necessary cut-off after 36 cycles, a user-friendly assay was established for simultaneous quantification of all three genes. The DNA was placed in separate wells but run using one common qPCR program. Although the inhibition tests were negative in this study, it is advisable to insert an internal amplification control (IAC) in the current qPCR method to detect false negative results in case of inhibition. As possible internal control a non-competitive IAC whereby different primers and probes and exogenous target DNA are added to the reaction mix in low copy numbers, may be used [34].

Using artificial contamination of cattle feces, a limit of quantification (LOQ) of <2.7 log copies g^−1^ feces was demonstrated for *stx1*, *stx2* (primer set b) and *eae;* the LOQ was 3.7 log copies g^−1^ feces for *stx2* (primer set a). This was due to the cut-off after 36 cycles. Taking the dilution factor of 160 into account, LOQ corresponded to 1 to 10 gene copies per reaction tube for *stx1*, *stx2* (primer set b) and *eae* and between 10 and 100 copies per reaction tube for *stx2* (primer set a). This means that the same high sensitivity was reached for STEC detection in cattle feces as for pure DNA in water. These results also indicate that all STEC DNA was recovered from the sample, and that no inhibiting compounds interfered with the real-time PCR reaction. Second, quantification results did not differ more than 1 log from the theoretically calculated number of genes in the sample.

To study the potential of the qPCR assay for detecting and quantifying STEC in cattle and on cattle farms, native cattle fecal samples were analyzed in parallel assays, using qPCR and a culture method as a reference. STEC was isolated from 10 animals. When we compared the genes carried by the isolates with the genes detected in the respective fecal sample by means of qPCR, much inconsistency was found. First, the genes carried by the strains obtained by means of the culture method (Table 4) did not correspond to the genes detected in the fecal sample (Figure 2). Further, from 15 *stx*-positive samples, no STEC strain could be isolated. These results demonstrated the high complexity of cattle fecal samples, with an abundance of microorganisms carrying the *stx* and *eae* genes. With qPCR it is measured if the genes *stx* or *eae* are somewhere present in the community DNA of the fecal sample, but the actual origin of the genes cannot be determined/Further in this section, we discuss the possible origin of the *stx* and *eae* signals detected with qPCR in the fecal sample.

In contrast to the discrepancies observed at the individual animal level, a clear correlation was observed between the detection of *stx* in the samples and the STEC-positive status of the farm. STEC was isolated on three out of six farms. The qPCR assay proved to be a valuable tool to detect culture-positive farms, as there was a good agreement between the two tests. In total, 83% of the cows identified as positive by the qPCR assay were indeed located on a culture-positive farm. In addition, 77% of the cows detected as negative by the qPCR assay originated from a culture-negative farm. Real-time PCR is fast and is not labor-intensive; this makes it suitable for use as a screening method. Due to the lack of common biochemical properties, STEC are difficult to distinguish from other *E. coli*. Culture methods generally isolate only a subset of serogroups [12,35,36], leaving other STEC serogroups undetected. Our qPCR method, used as a predictive tool, makes use of a pool of *stx* genes present in the fecal sample to identify potential high-risk farms. Thus far, no explanation for this pool of *stx* genes on STEC-positive farms has been given. No STEC could be isolated from many *stx*-positive samples. The *stx* genes may originate from either STEC cells of serogroups other than those selected by the culture method, non-*E. coli* bacteria, like *Shigella dysenteriae*, *stx*-phages, or free DNA molecules. Intensive culture methods [11,37], *stx*-phage isolation methods and molecules which exclude the detection of free DNA [38] may give insight into the actual origin of the *stx* genes that were detected in this study. Currently, the hypothesis can be made that once STEC cells proliferate on a farm, a wide variation of *E. coli* becomes transfected by *stx*-phages, which exponentially multiply the population of STEC cells on the farm. If this is true, then many more other questions arise, such as what is their stability, why would not all *E. coli* become STEC, and what is their importance in human pathogenicity? More research is needed to identify this pool of *stx* genes and to identify their role in the epidemiology of STEC on cattle farms.

The aim of this study was to investigate if quantitative data obtained by qPCR give additional information about the degree of contamination of the individual animal or farm. Validation using artificially inoculated cattle feces confirmed that the assay is perfectly suitable for quantification of *stx1*, *stx2* and *eae*. Unfortunately, the research question itself was answered negatively, because quantitative data did not give additional insights into the STEC contamination level of the animal or the farm. We observed that *stx* levels were equally high in culture-negative and culture-positive cattle. This was in contrast to results described by Ibekwe *et al.* [17], which proved the potential of qPCR to quantify STEC O157 in naturally contaminated cattle feces, and promoted a culture-free approach. However, the latter authors might not have looked at the level of *stx* in culture-negative animals on the farm, which according to our findings would have contained high levels of *stx* as well.

On the other hand, the presence of both *stx* and *eae* genes as detected by qPCR in an individual animal or on a farm was not correlated to the isolation of *eae*-harboring STEC strains by means of the culture method in the animal or farm in question. The explanation is that using DNA-based methods, it cannot be determined whether genes are present within one cell or not. *Eae*-harboring organisms other than STEC produce *eae* signals undistinguishable from *eae*-harboring STEC. The *eae* genes may originate from EPEC cells, other species, such as *Citrobacter rodentium* and *Escherichia albertii*, or free DNA molecules. In the literature, the presence of EPEC and STEC on the same cattle farm has been described [39,40].

## 4. Experimental Section

### 4.1. Method Development and Testing

#### 4.1.1. Bacterial Isolates

The bacterial strains used in this study are listed in Table 1. All strains were stored at −80 °C using Pro-Lab Microbank cryovials (Pro-Lab, Richmond Hill, ON, Canada) according to the manufacturer’s instructions. Strains were cultured on Tryptone Soy Agar (TSA; Oxoid Ltd., Basingstoke, Hampshire, UK) and incubated either aerobically or anaerobically, as appropriate, at 37 °C for 24 h, except for *Campylobacter,* which was incubated under microaerophilic conditions (at 5% O_2_, 10% CO_2_, 85% N_2_ in an O_2_/CO_2_ incubator; Thermo Forma, OH, USA) at 42 °C for 48 h. 

For artificial inoculation of cattle feces, strains MB3936 (O26 *stx1*^+^ *stx2*^+^ *eae*^+^) and MB4378 (O138 *stx2e^+^*) were grown by transferring one colony from TSA into Tryptone Soy Broth (TSB; Oxoid), and incubating at 37 °C for 24 h. Then, the stationary grown culture was ten-fold serially diluted in Buffered Peptone Water (BPW; Oxoid) at 4 °C. Inoculation of fecal samples was performed immediately, as well as the determination of the number of total number of viable cells. The latter was determined on TSA inoculated with 0.1 mL of 10^−6^ to 10^−8^ dilutions in duplicate and incubated at 37 °C overnight.

#### 4.1.2. Cattle Fecal Samples

##### Sample Preparation and DNA Extraction

Sixty cattle fecal samples were taken from 10 individual adult animals at six local farms (beef, dairy and combined farms). One STEC culture-negative sample (determined using the culture method; see below) was used for artificial inoculation to evaluate the qPCR assays. The other 59 samples were analyzed as native fecal samples using qPCR and classical culture for STEC isolation. For artificial inoculation (see below), 25 g subsamples were diluted tenfold in 225 mL TSB (Oxoid) in a filter stomacher bag. Subsequently, 2.5 mL volumes were concentrated by centrifugation (14,000 g, for 5 min) and the pellet (corresponding with 0.25 g feces) was subjected to DNA extraction using the QIAamp DNA Stool Mini Kit (Qiagen Inc, Valencia, CA, US) according to the manufacturer’s instructions. DNA was finally dissolved in a volume of 200 µL of elution supplied in the kit buffer. For analysis of the native samples, 0.25 g of each sample was subjected to DNA extraction by using the QIAamp DNA Stool Mini Kit (Qiagen Inc) according to the manufacturer’s instructions. qPCR analysis was performed on the DNA samples, and fecal samples were not enriched before processing. Additionally, from each native fecal sample, 25 g was diluted tenfold in 225 mL TSB (Oxoid) in a filter stomacher bag and subjected to the classical culture method for STEC isolation (see below).

##### Artificial Contamination with STEC

The sample used for artificial contamination was divided into 22, 25 g subsamples that were diluted separately tenfold in 225 mL TSB (Oxoid) in a filter stomacher bag by stomaching for 2 min. Appropriate volumes of diluted bacterial cultures were added to an individual subsample to obtain 16 contamination levels of STEC strain MB3936, ranging between 5.0 × 10^7^ and 1.6 × 10^2^ CFU g^−1^ feces and six contamination levels of STEC strain MB4378, ranging between 1.0 × 10^7^ and 2.7 × 10^2^ CFU g^−1^ feces. One subsample was not inoculated and was used as a blank sample.

#### 4.1.3. Real-Time PCR (qPCR)

##### Preparation of DNA Standards

DNA standards were made for STEC strain MB3936 and for STEC strain MB4378. Isolated DNA was serially diluted tenfold in water and in a series from 10^5^ to 10 copies used as standard in the qPCR. DNA isolation was performed according to the method described by Flamm *et al.* [41]. The concentration and purity of the purified DNA was determined by measuring the optical density by photo spectroscopy at 260 nm using the Nanodrop^®^ ND-1000 UV-VIS Spectrophotometer (Nanodrop Technologies, Wilmington, NC, USA). The number of genomic copies was calculated using the equation M = n × 1.093 × 10^−21^g bp^−1^ with M as the mass of one genome and n as the total number of base pairs (bp). For *E. coli* strain O157:H7 EDL933, this was determined to be 5.53 × 10^6^ bp [42].

##### Primers and Probes

Gene sequences of the different variants of *stx1*, *stx2* and *eae* were downloaded from the EMBL/Genbank database using BLAST (based on sequence similarity). Subsequently, the sequences were aligned using Kodon software version 3.5 (Applied Maths NV, Sint-Martens-Latem, Belgium). The local database in Kodon contained 25, 137 and 82 gene sequences of *stx1*, *stx2* and *eae*, respectively, which were grouped into subtypes: (1) *stx1a*, *stx1c* and *stx1d*; *stx2a*, *stx2b*, *stx2c*, *stx2d*, *stx2e*, *stx2f* and *stx2g*; (2) *eae α1*, *eae β1*, *eae γ1*, *eae γ2*, *eae ε*, *eae ζ*; and (3) some other variants of *eae*. Subtypes of *stx* were denominated according to the subtyping nomenclature established at the 7th International Symposium on Shiga Toxin (Verocytotoxin)-Producing *Escherichia coli* Infections (Buenos Aires, 10–13 May, 2009). Various primer-probe combinations for *stx1*, *stx2* and *eae* from the literature [17,22,23,24,25,26,27,28,29,30,31,32,33] and newly designed primers and probes using Primer Express 2.0 (Applied Biosystems, Foster City, CA, US), were aligned to the local database. The combinations that resulted in the fewest mismatches (≤2 in a primer, ≤1 in a probe) for the aforementioned subtypes (except *stx2f*) were selected. Occurring mismatching bases were replaced by inosine. Selected primer sets for *stx1* quantification included primer set stx1-F/-R (5’-gacgcagtctgtigcaagag-3’/5’-cgaaaacgiaaagcttcagctg-3’), designed in this study, and primer sets 598-F/1015’-R (5’-agtcgtacggggatgcagataaat-3’/5’-icggicacatagaaggaaactcat-3’) and 598-F/1015”-R (5’-agtcgtacggggatgcagataaat- 3’/5’-cggicacatagaaggaaactcat-3’), both adapted from Bellin *et al.* [32]. Selected primer sets for *stx2* quantification included primer set stx2-F/-R (5’-tcaggcaiatacagagagaatttcg-3’/5’-ccggigtcatcgtataiacag-3’), designed in this study, and primer sets stx2-F/-R_a_ and stx2-F/-R_b_, which contained the same forward primer and a slightly adapted reverse primer stx2-R_a_ (5’-ccggigtcatcgtatacacag-3’) or stx2-R_b_ (5’-ccggigtcatcgtataaacag-3’) containing only one inosine base instead of two. In addition, primer set Fitz-F/-R (5’-ggcactgtctgaaactgctcc-3’/5’-tcgccaittatctgacattctg-3’), adapted from Fitzmaurice *et al.* [33], was evaluated for *stx2* quantification. Selected primer sets for *eae* quantification comprised primer set eae-F/-R (5’-ggaagccaaagcgcacaa-3’/5’-ggcicgagcigtcactttataa-3’) and primer set eae-F/-R_2_ using an adapted reverse primer eae-R_2_ (5’-cggtcataggcgcgagc-3’), both designed in this study. The utility of the selected primers was evaluated in a qPCR assay using SYBR Green fluorescence (see further).

The finally selected primers with corresponding hydrolysis probes are listed in Table 1. Two primer sets were needed to enclose all subtypes of *stx2,* except *stx2f*, without mismatching bases. Set a consists of primers stx2-F and stx2-R_a_ and probe stx2-P. Set b consists of primers stx2-F and stx2-R_b_ and probe stx2-P. The hydrolysis probes stx1-P and stx2-P carried a minor groove-binding non-fluorescent quencher (MGBNFQ) in a combination with a 6-carboxyfluorescin (FAM) fluorescent label (Applied Biosystems). Probe eae-P carried a black hole quencher (BHQ-1) and a FAM fluorescent label (Eurogentec, Seraing, Belgium).

##### qPCR Using SYBR Green Fluorescence

The utility of the selected primers was evaluated based on the C_q_ values (threshold cycle) in a qPCR using SYBR Green I and DNA standards of strains MB3936 and MB4378 using 10-fold serial dilutions of 10^5^ to 10. The qPCR was carried out in a 25 µL volume containing 1 × SYBR Green I Master Mix (Applied Biosystems), primers (final concentration 600 nM of each primer; Eurogentec, Seraing, Belgium) and 5 µL of template DNA. The qPCR was performed on a LightCycler^®^ 480 (Roche Diagnostics) using the LightCycler^®^ 480 software, with the following program: activation of the enzyme at 95 °C for 10 min, followed by 40 cycles of 95 °C for 15 s and 60 °C for 1 min. Finally, melting curve analysis of the PCR products was performed by completing one additional amplification cycle and gradually increasing the temperature from 60 °C to 95 °C. The program was ended after a cooling at 40 °C for 30 s.

##### qPCR Using Hydrolysis Probes

The three genes were quantified using four qPCR assays (1 × *stx1*, 2 × *stx2*, 1 × *eae*) in separate wells of the same plate. The qPCR assays were carried out in a 25 µ volume containing 1 × TaqMan^®^ Environmental Master Mix 2.0 (Applied Biosystems), primers and probe designed in this study (final concentration 300 nM of each primer and 100 nM probe; Eurogentec) and 5 µL template DNA of the standards MB3936 or MB4378. qPCR was performed with the following amplification program: initial activation of the enzyme at 95 °C for 5 min followed by 40 cycles of 95 °C for 15 s and 1 min annealing and elongation at 60 °C, and cooling at 40 °C for 30 s.

DNA standards were used as a template to create standard curves. The amplification efficiency (E) was calculated as E = (10^(−1/sl^°^pe)^ − 1) × 100% [43]. The linear correlation coefficient R^2^ represented the linearity.

Inclusivity, defined as the detection of target strains [44], was tested with 23 *E. coli* strains carrying a wide variation of *stx1*, *stx2* or *eae* genes, whereas exclusivity, defined as the non-detection of non-target strains [44], was assessed with 22 non-*E. coli* and non-pathogenic *E. coli* strains (Table 5)

##### Quantification of STEC Virulence Genes in an Artificially Contaminated Cattle Fecal Sample

Inhibition by the fecal matrix was evaluated by performing the qPCR assays for *stx1*, *stx2* and *eae* on eight cattle fecal samples originating from different farms. The samples had previously been confirmed negative by the qPCR and culture method. For the inhibition test, diluted genomic DNA from strain MB3936 was added to the PCR reaction, in order to obtain 10^4^ gene copies per reaction tube of genes *stx1*, *stx2* and *eae*. An increase of the Cq as compared to the standard curve would indicate that inhibition occurred.

From one cattle fecal sample, 16 subsamples were inoculated with different levels of strain MB3936, six subsamples with various levels of strain MB4378 and one subsample was not inoculated (blank). The fecal sample chosen for artificial inoculation had previously been confirmed as culture-negative for STEC, and did not generate positive signals for *stx1*, *stx2* or *eae* with qPCR. *stx1*, *stx2* and *eae* gene copies were quantified (primers and probes in Table 2) in fecal subsamples inoculated with strain MB3936 and in the blank sample. The samples inoculated with this strain (MB3936) were analyzed using primer-probe set a (stx2-F/-P/-R_a_) to quantify *stx2* variants containing the polymorphism which matches with primer-probe set a. Fecal subsamples inoculated with strain MB4378 and the blank sample were analyzed using primer-probe set b (stx2-F/-P/-R_a_) for the quantification of *stx2* variants which match with primer-probe set b. To quantify STEC virulence genes in feces, DNA standards of strains MB3936 and MB4378 were included in the same qPCR run. Based on the observed C_q_ values, gene copy numbers in 1 g inoculated feces were calculated while accounting for the dilution factor in the qPCR (×160). Quantification results were compared with theoretically calculated numbers of genes in the sample based on the inocula. The limit of quantification (LOQ) was defined as the lowest number of organisms that can be quantified in the fecal sample (1 g).

### 4.2. Study of the qPCR Assays on Native Cattle Feces Samples

#### 4.2.1. Quantification of STEC Virulence Genes in Native Fecal Samples

For 59 native cattle fecal samples, the qPCR assays for quantification of genes *stx1*, *stx2* and *eae* were carried out. For quantification of *stx2* both primer sets a and b were performed, because the type of *stx2* in a naturally contaminated sample is unknown and therefore also the set that will match 100%. Gene copy numbers in the reaction were calculated based on the DNA standard of strain MB3936 for enumeration of *stx1*, *stx2* (enumeration of *stx2* variants which match with primer-probe set a) and *eae*. For the enumeration of *stx2* variants which match with primer-probe set b, the DNA standard of strain MB4378 was used. Subsequently, gene copy numbers in 1 g feces were calculated (dilution factor 160).

Cattle fecal samples for which *stx1* or *stx2* could be quantified were regarded as *stx*-positive. On the farm level, *stx*-positive animals were counted to determine the farm prevalence.

#### 4.2.2. Classical Culture for Isolation of STEC

The same tenfold diluted and homogenized native cattle fecal samples were subjected to the STEC isolation method as described by Possé *et al.* [13] for five important serogroups (O157, O26, O103, O111, O145). Briefly, 8 mg L^−1^ novobiocine (Sigma, St. Louis, MO, USA), 16 mg L^−1^ vancomycin (Sigma), 2 mg L^−1^ rifampicin (Sigma), 1.5 g L^−1^ bile salts (Oxoid) and 1.0 mg L^−1^ potassium tellurite (Sigma) were supplemented to TSB (Biorad) to prepare the enrichment broth and incubation was performed for 24 h at 42 °C. *Post* incubation, the enrichment broths were spread on a O157 agar plate and a non-O157 agar plate. In parallel, immunomagnetic separation (IMS) was performed on 1 mL of the enrichment broth, using Dynabeads (Invitrogen) for serogroups O26, O103 and O157 and using Captivate beads (Lab M, Bury, UK) for O111 and O145, followed by plating of the resulting solution (100 µL) on the O157 and non-O157 agar plates. Plates were incubated at 37 °C for 24 h. Colonies were evaluated based on their general appearance and color. All suspected colonies according to the description of Possé *et al.* [45] with a maximum of 10 colonies per plate were evaluated with a multiplex PCR for *stx1*, *stx2*, and *eae* [46]. Subsequently, *stx*- or *eae*-harboring isolates were subjected to serogroup PCR for O26 [47], O103 [48], O111 and O157 [49], O145 [50], O91 [51], and O121 [52].

Individual animals for which STEC could be isolated from their fecal sample were considered culture-positive. Farms which harbored culture-positive samples were considered culture-positive farms.

### 4.3. Statistical Analysis

McNemar’s test was used to check agreements between the results of the qPCR assay and the results of the culture method. A kappa value was computed as a measure of agreement between the two tests. The specificity and sensitivity of all qPCR assays were computed as:

Specificity = True Negative/(True Negative + False Positive) × 100 (1)

Sensitivity = True Positive/(True Positive + False Negative) × 100 (2)

To test if the prevalence of *stx* was significantly higher on the culture-positive farms (A, B, C) than on the culture-negative farms (D, E, F), a Mann–Whitney rank test was performed.

Statistical analysis was performed using SPSS 19.0 (IBM, Chicago, IL, USA). The significance level α was set at 0.05.

## 5. Conclusions

In conclusion, we have established a sensitive method to quantify STEC virulence genes *stx1*, *stx2*, and *eae* in cattle feces, including all variants of the genes which have been implicated in human infection. Moreover, this study has demonstrated that STEC culture-positive farms had moderate to significantly higher prevalences of *stx* compared to culture-negative farms. Consequently, the qPCR assay may serve as a fast screening tool to identify potential high-risk farms. Quantitative data did not yield additional insight into the contamination level of the animal or the farm. Finally, the presence of both *stx* and *eae* genes in the same cattle fecal sample or farm as detected with qPCR was not correlated to the presence of *eae*-harboring STEC strains isolated with the culture method from the respective animal or farm. More research is needed to confirm these findings and to define criteria to distinguish potential high risk farms.

## References

[1] Beutin L., Krause G., Zimmermann S., Kaulfuss S., Gleier K. (2004). Characterization of Shiga toxin-producing *Escherichia coli* strains isolated from human patients in Germany over a 3-year period. J. Clin. Microbiol..

[2] Karmali M.A. (1989). Infection by verocytotoxin-producing *Escherichia coli*. Clin. Microbiol. Rev..

[3] Vaz T.M., Irino K., Nishimura L.S., Cergole-Novella M.C., Guth B.E. (2006). Genetic heterogeneity of Shiga toxin-producing *Escherichia coli* strains isolated in Sao Paulo, Brazil, from 1976 through 2003, as revealed by pulsed-field gel electrophoresis. J. Clin. Microbiol..

[4] Verstraete K., de Reu K., van Weyenberg S., Piérard D., de Zutter L., Herman L., Robyn J., Heyndrickx M. (2012). Genetic characteristics of Shiga toxin-producing *E. coli* O157, O26, O103, O111, and O145 isolates from humans, food, and cattle in Belgium. Epidemiol. Infect..

[5] Ramachandran V., Brett K., Hornitzky M.A., Dowton M., Bettelheim K.A., Walker M.J., Djordjevic S.P. (2003). Distribution of intimin subtypes among *Escherichia coli* isolates from ruminant and human sources. J. Clin. Microbiol..

[6] Persson S., Olsen K.E.P., Ethelberg S., Scheutz F. (2007). Subtyping method for *Escherichia coli* Shiga toxin (verocytotoxin) 2 variants and correlations to clinical manifestations. J. Clin. Microbiol..

[7] Boerlin P. (1999). Evolution of virulence factors in Shiga-toxin-producing *Escherichia coli*. Cell. Mol. Life Sci..

[8] Bettelheim K.A. (2003). Non-O157 verotoxin-producing *Escherichia coli*: A problem, paradox, and paradigm. Exp. Biol. Med..

[9] Blanco M., Padola N.L., Kruger A., Sanz M.E., Blanco J.E., Gonzalez E.A., Dahbi G., Mora A., Bernardez M.I., Etcheverria A.I. (2004). Virulence genes and intimin types of Shiga-toxin-producing *Escherichia coli* isolated from cattle and beef products in Argentina. Int. Microbiol..

[10] European Food Safety Authority (EFSA) (2011). The European Union summary report on trends and sources of zoonoses, zoonotic agents and food-borne outbreaks in the in 2009. EFSA J..

[11] Fukushima H., Seki R. (2004). High numbers of Shiga toxin-producing *Escherichia coli* found in bovine faeces collected at slaughter in Japan. FEMS Microbiol. Lett..

[12] Verstraete K., de Zutter L., Messens W., Herman L., Heyndrickx M., de Reu K. (2010). Effect of the enrichment time and immunomagnetic separation on the detection of Shiga toxin-producing *Escherichia coli* O26, O103, O111, O145 and sorbitol positive O157 from artificially inoculated cattle faeces. Vet. Microbiol..

[13] Possé B., de Zutter L., Heyndrickx M., Herman L. (2008). Quantitative isolation efficiency of O26, O103, O111, O145 and O157 STEC serotypes from artificially contaminated food and cattle feces samples using a new isolation protocol. J. Appl. Microbiol..

[14] Arthur T.M., Keen J.E., Bosilevac J.M., Brichta-Harhay D.M., Kalchayanand N., Shackelford S.D., Wheeler T.L., Nou X., Koohmaraie M. (2009). Longitudinal study of *Escherichia coli* O157:H7 in a beef cattle feedlot and role of high-level shedders in hide contamination. Appl. Environ. Microbiol..

[15] Fratamico P.M., Bagi L.K., Pepe T. (2000). A multiplex polymerase chain reaction assay for rapid detection and identification of *Escherichia coli* O157:H7 in foods and bovine feces. J. Food Prot..

[16] Ibekwe A.M., Grieve C.M. (2003). Detection and quantification of *Escherichia coli* O157:H7 in environmental samples by real-time PCR. J. Appl. Microbiol..

[17] Ibekwe A.M., Watt P.M., Grieve C.M., Sharma V.K., Lyons S.R. (2002). Multiplex fluorogenic real-time PCR for detection and quantification of *Escherichia coli* O157:H7 in dairy wastewater wetlands. Appl. Environ. Microbiol..

[18] Jacob M.E., Shi X., An B., Nagaraja T.G., Bai J. (2012). Evaluation of a multiplex real-time polymerase chain reaction for the quantification of *Escherichia coli* O157 in cattle feces. Foodborne Pathog. Dis..

[19] Isobe J., Kimata K., Shimojima M., Hosorogi S., Tanaka D., Gyobu Y. (2004). Isolation of *Escherichia coli* O128:HNM harboring *stx2f* gene from diarrhea patients. Kansenshogaku Zasshi.

[20] Kawano K., Okada M., Haga T., Maeda K., Goto Y. (2008). Relationship between pathogenicity for humans and stx genotype in Shiga toxin-producing *Escherichia coli* serotype O157. Eur. J. Clin. Microbiol. Infect. Dis..

[21] Werbrouck H., Botteldoorn N., Uyttendaele M., Herman L., van Coillie E. (2007). Quantification of gene expression of *Listeria monocytogenes* by real-time reverse transcription PCR: Optimization, evaluation and pitfalls. J. Microbiol. Methods.

[22] Belanger S.D., Boissinot M., Menard C., Picard F.J., Bergeron M.G. (2002). Rapid detection of Shiga toxin-producing bacteria in feces by multiplex PCR with molecular beacons on the smart cycler. J. Clin. Microbiol..

[23] Fratamico P.M., DebRoy C., Miyamoto T., Liu Y.H. (2009). PCR Detection of Enterohemorrhagic *Escherichia coli* O145 in food by targeting genes in the *E. coli* O145 O-Antigen gene cluster and the Shiga Toxin 1 and Shiga Toxin 2 genes. Foodborne Pathog. Dis..

[24] Grys T.E., Sloan L.M., Rosenblatt J.E., Patel R. (2009). Rapid and sensitive detection of Shiga toxin-producing *Escherichia coli* from nonenriched stool specimens by real-time PCR in comparison to enzyme immunoassay and culture. J. Clin. Microbiol..

[25] Iijima Y., Asako N.T., Aihara M., Hayashi K. (2004). Improvement in the detection rate of diarrhoeagenic bacteria in human stool specimens by a rapid realtime PCR assay. J. Med. Microbiol..

[26] Jinneman K.C., Yoshitomi K.J., Weagant S.D. (2003). Multiplex real-time PCR method to identify Shiga toxin genes *stx1* and *stx2* and *Escherichia coli* O157:H7/H- serotype. Appl. Environ. Microbiol..

[27] O’Hanlon K.A., Catarame T.M.G., Duffy G., Blair I.S., McDowell D.A. (2004). Rapid detection and quantification of *E. coli* O157/O26/O111 in minced beef by real-time PCR. J. Appl. Microbiol..

[28] Perelle S., Dilasser F., Grout J.L., Fach P. (2004). Detection by 5’-nuclease PCR of Shiga-toxin producing *Escherichia coli* O26, O55, O91, O103, O111, O113, O145 and O157:H7, associated with the world's most frequent clinical cases. Mol. Cell. Probes.

[29] Reischl U., Youssef M.T., Kilwinski J., Lehn N., Zhang W.L., Karch H., Strockbine N.A. (2002). Real-time fluorescence PCR assays for detection and characterization of Shiga toxin, intimin, and enterohemolysin genes from Shiga toxin-producing *Escherichia coli.*. J. Clin. Microbiol..

[30] Sharma V.K., Dean-Nystrom E.A., Casey T.A. (1999). Semi-automated fluorogenic PCR assays (TaqMan) for rapid detection of *Escherichia coli* O157:H7 and other Shiga toxigenic *E. coli*. Mol. Cell. Probes.

[31] Sharma V.K. (2002). Detection and quantitation of enterohemorrhagic *Escherichia coli* O157, O111, and O26 in beef and bovine feces by real-time polymerase chain reaction. J. Food Prot..

[32] Bellin T., Pulz M., Matussek A., Hempen H.G., Gunzer F. (2001). Rapid detection of enterohemorrhagic *Escherichia coli* by real-time PCR with fluorescent hybridization probes. J. Clin. Microbiol..

[33] Fitzmaurice J., Glennon M., Duffy G., Sheridan J.J., Carroll C., Maher M. (2004). Application of real-time PCR and RT-PCR assays for the detection and quantitation of *VT1* and *VT2* toxin genes in *E. coli* O157:H7. Mol. Cell. Probes.

[34] Hoorfar J., Malorny B., Abdulmawjood A., Cook N., Wagner M., Fach P. (2004). Practical considerations in design of internal amplification controls for diagnostic PCR assays. J. Clin. Microbiol..

[35] Verstraete K., de Zutter L., Robyn J., Daube G., Herman L., Heyndrickx M., de Schaetzen M.A., de Reu K. (2012). Validation of a method for simultaneous isolation of Shiga toxin-producing *Escherichia coli* O26, O103, O111 and O145 from minced beef by an international ring-trial. Foodborne Pathog. Dis..

[36] Verstraete K., Robyn J., Del-Favero J., de Rijk P., Joris A., Herman L., Heyndrickx M., de Zutter L., de Reu K. (2012). Evaluation of a multiplex-PCR detection in combination with an isolation method for STEC O26, O103, O111, O145 and sorbitol fermenting O157 in food. Food Microbiol..

[37] Menrath A., Wieler L.H., Heidemanns K., Semmler T., Fruth A., Kemper N. (2010). Shiga toxin producing *Escherichia coli*: Identification of non-O157:H7-super-shedding cows ans related risk factors. Gut Pathog..

[38] Bae S., Wuertz S. (2009). Discrimination of viable and dead fecal Bacteroidales bacteria by quantitative PCR with propidium monoazide. Appl. Environ. Microbiol..

[39] Hornitzky M.A., Mercieca K., Bettelheim K.A., Djordjevic S.P. (2005). Bovine feces from animals with gastrointestinal infections are a source of serologically diverse atypical enteropathogenic *Escherichia coli* and Shiga toxin-producing *E. coli* strains that commonly possess intimin. Appl. Environ. Microbiol..

[40] Blanco M., Schumacher S., Tasara T., Zweifel C., Blanco J.E., Dahbi G., Blanco J., Stephan R. (2005). Serotypes, intimin variants and other virulence factors of eae positive *Escherichia coli* strains isolated from healthy cattle in Switzerland. Identification of a new intimin variant gene (eae-eta2). BMC Microbiol..

[41] Flamm R.K., Hinrichs D.J., Thomashow M.F. (1984). Introduction of Pam-Beta-1 into *Listeria monocytogenes* by conjugation and homology between native *L. monocytogenes* plasmids. Infect. Immun..

[42] Perna N.T., Plunkett G., Burland V., Mau B., Glasner J.D., Rose D.J., Mayhew G.F., Evans P.S., Gregor J., Kirkpatrick H.A. (2001). Genome sequence of enterohaemorrhagic *Escherichia coli* O157:H7. Nature.

[43] Knutsson R., Lofstrom C., Grage H., Hoorfar J., Radstrom P. (2002). Modeling of 5’ nuclease real-time responses for optimization of a high-throughput enrichment PCR procedure for *Salmonella enterica*. J. Clin. Microbiol..

[44] International Organization for Standardization (ISO) ISO 16140: Microbiology of Food and Animal Feeding Stuffs—Protocol for the Validation of Alternative Methods. International Organisation for Standardisation.

[45] Possé B., de Zutter L., Heyndrickx M., Herman L. (2008). Novel differential and confirmation plating media for Shiga toxin-producing *Escherichia coli* serotypes O26, O103, O111, O145 and sorbitol-positive and -negative O157. FEMS Microbiol. Lett..

[46] Botteldoorn N., Heyndrickx M., Rijpens N., Herman L. (2003). Detection and characterization of verotoxigenic *Escherichia coli* by a VTEC/EHEC multiplex PCR in porcine faeces and pig carcass swabs. Res. Microbiol..

[47] DebRoy C., Roberts E., Kundrat J., Davis M.A., Briggs C.E., Fratamico P.M. (2004). Detection of *Escherichia coli* serogroups O26 and O113 by PCR amplification of the *wzx* and *wzy* genes. Appl. Environ. Microbiol..

[48] Fratamico P.M., DebRoy C., Strobaugh T.P., Chen C.Y. (2005). DNA sequence of the *Escherichia coli* O103O antigen gene cluster and detection of enterohemorrhagic *E. coli* O103 by PCR amplification of the *wzx* and *wzy* genes. Can. J. Microbiol..

[49] Paton A.W., Paton J.C. (1998). Detection and characterization of shiga toxigenic *Escherichia coli* by using multiplex PCR assays for *stx*(*1*), *stx*(*2*), *eaeA*, enterohemorrhagic *E-coli hlyA*, *rfb*(*O111*), and *rfb*(*O.157*). J. Clin. Microbiol..

[50] Feng L., Senchenkova S.N., Tao J., Shashkov A.S., Liu B., Shevelev S.D., Reeves P.R., Xu J.G., Knirel Y.A., Wang L. (2005). Structural and genetic characterization of enterohemorrhagic *Escherichia coli* O145O antigen and development of an O145 serogroup-specific PCR assay. J. Bacteriol..

[51] Perelle S., Dilasser F., Grout J., Fach P. (2002). Identification of the O-antigen biosynthesis genes of *Escherichia coli* O91 and development of a O91 PCR serotyping test. J. Appl. Microbiol..

[52] Fratamico P.M., Briggs C.E., Needle D., Chen C.Y., DebRoy C. (2003). Sequence of the Escherichia coli O121 O-antigen gene cluster and detection of enterohemorrhagic *E. coli* O121 by PCR amplification of the wzx and wzy genes. J. Clin. Microbiol..

